# Explainable machine learning model for predicting decline in platelet count after interventional closure in children with patent ductus arteriosus

**DOI:** 10.3389/fped.2025.1519002

**Published:** 2025-02-06

**Authors:** Song-Yue Zhang, Yi-Dong Zhang, Hao Li, Qiao-Yu Wang, Qiao-Fang Ye, Xun-Min Wang, Tian-He Xia, Yue-E He, Xing Rong, Ting-Ting Wu, Rong-Zhou Wu

**Affiliations:** ^1^ Children's Heart Center, The Second Affiliated Hospital and Yuying Children's Hospital of Wenzhou Medical University, Wenzhou, China; ^2^ Fujian Children's Hospital, Fujian, China

**Keywords:** children, decline in platelet count, interventional closure, machine learning, patent ductus arteriosus

## Abstract

**Background:**

This study aimed to apply four machine learning algorithms to develop the optimal model to predict decline in platelet count (DPC) after interventional closure in children with patent ductus arteriosus (PDA).

**Methods:**

Data from children with PDA who underwent successful transcatheter closure at the Second Affiliated Hospital of Wenzhou Medical University and Yuying Children's Hospital from January 2016, to December 2022, were collected. The cohort data were split into training and testing sets. DPC following the intervention is defined as a percentage DPC ≥25% [(baseline platelet count−nadir platelet count)/baseline platelet count]. The extra tree algorithm was used for feature selection and four ML algorithms [random forest (RF), adaptive boosting, extreme gradient boosting, and logistic regression] were established. Moreover, SHapley Additive exPlanation (SHAP) to explain the importance of features and the ML models.

**Results:**

This study included 330 children who underwent successful transcatheter closure of PDA, of which 113 (34.2%) experienced DPC. After 62 clinical features were considered, the extra tree algorithm selected six clinical features to build the ML models. Amongst the four ML algorithms, the RF model achieved the greatest AUC. SHAP analysis revealed that pulmonary artery systolic pressure, size of defect and weight were the top three most important clinical features in the RF model. Furthermore, clinical descriptions of two children with PDA, with accurate predictions, and explanations of the prediction results were provided.

**Conclusion:**

In this study, an ML model (RF) capable of predicting post-intervention DPC in children with PDA undergoing transcatheter closure was established.

## 1 Introduction

Patent ductus arteriosus (PDA) is one of the most common congenital heart diseases. Transcatheter closure has become the preferred method for treating PDA due to its non-invasive nature, low risk, rapid recovery and reliable efficacy (1, 2). As surgical techniques continue to advance, postoperative complications, such as decline in platelet count (DPC), arrhythmias, haemolysis, device dislodgment and detachment, have received increasing attention. Mild cases of DPC may lead to gingival or skin bleeding and severe cases can result in significant visceral bleeding and even life-threatening situations (3, 4). Therefore, early identification of children with PDA at risk of post-intervention DPC is of paramount importance for clinicians.

In recent years, with the development of artificial intelligence, machine learning (ML) has been increasingly applied in clinical research (5). ML can analyse and interpret large volumes of data, thereby enhancing disease diagnosis, prediction and treatment outcomes. In contrast to conventional statistical analysis techniques, machine learning has the capability to scrutinize intricate nonlinear connections and uncover previously undiscovered associations, thereby delivering more profound insights into clinical data (6). For instance, in a study on malnutrition following congenital heart disease surgery, the development of an explainable ML model exhibited superior performance in predicting malnutrition in children with congenital heart disease 1 year postoperatively, Consequently, facilitating clinicians in formulating personalized therapeutic and dietary monitoring approaches (7). However, despite the excellent predictive accuracy of ML models, their practical clinical application is limited due to the “black-box problem”, where the decision-making process of ML models is opaque, making the results difficult to interpret (8, 9).

In this study, an ML model was developed to predict whether children with PDA would experience DPC after undergoing transcatheter closure. SHapley Additive explanation (SHAP) was used to interpret the ML model to address its “black-box problem” (10, 11), enabling clinicians to gain an enhanced understanding of the decision-making process, predictions and outcomes of the model and take timely intervention measures.

## 2 Methods

### 2.1 Data source and population

A total of 333 children with PDA who underwent successful transcatheter closure at the Second Affiliated Hospital of Wenzhou Medical University and Yuying Children's Hospital from January 2016, to December 2022, were included in this study. All children provided informed consent and exclusion criteria were applied as follows: (1) concomitant bleeding disorders or haematological diseases, such as aplastic anaemia; (2) concomitant other types of congenital heart diseases requiring surgical intervention; (3) history of preoperative heparin use or long-term antiplatelet drug use; (4) concomitant infective endocarditis or other uncontrolled infections; (5) baseline platelet count <100 × 10^9^/L. Data was gathered from the subsequent two origins and employed as early prognostic markers: preoperative and intraoperative databases. The study variables encompassed demographic characteristics, clinical factors, laboratory tests and ancillary examinations.

The cohort data was further randomly divided into two parts: the training set accounted for 70% and the test set for 30%. The model was trained on the training set and hyperparameter tuning was performed using extra tree algorithm (12).

### 2.2 Outcome variables

Currently, the definition of post-intervention DPC in congenital heart disease can be categorised into three main groups. The first category is based on the absolute value of postoperative platelet count, classifying it as mild DPC (100–150 × 10^9^/L), moderate DPC (50–100 × 10^9^/L) or severe DPC (<50 × 10^9^/L). The second category involves determining the percentage DPC by using the following formula: (baseline platelet count - nadir platelet count)/baseline platelet count × 100. In this category, no DPC is defined as <10%, mild DPC as 10%–49%, and severe DPC as ≥50%. The third category defines DPC as percentage DPC25%, which has been shown to better reflect the actual occurrence of DPC following transcatheter closure (13). Therefore, in the present study, the definition of DPC as percentage DPC ≥25% (DPC) was adopted and <25% (NO-DPC) indicated absence of DPC.

### 2.3 Feature extraction

**Figure 1:**
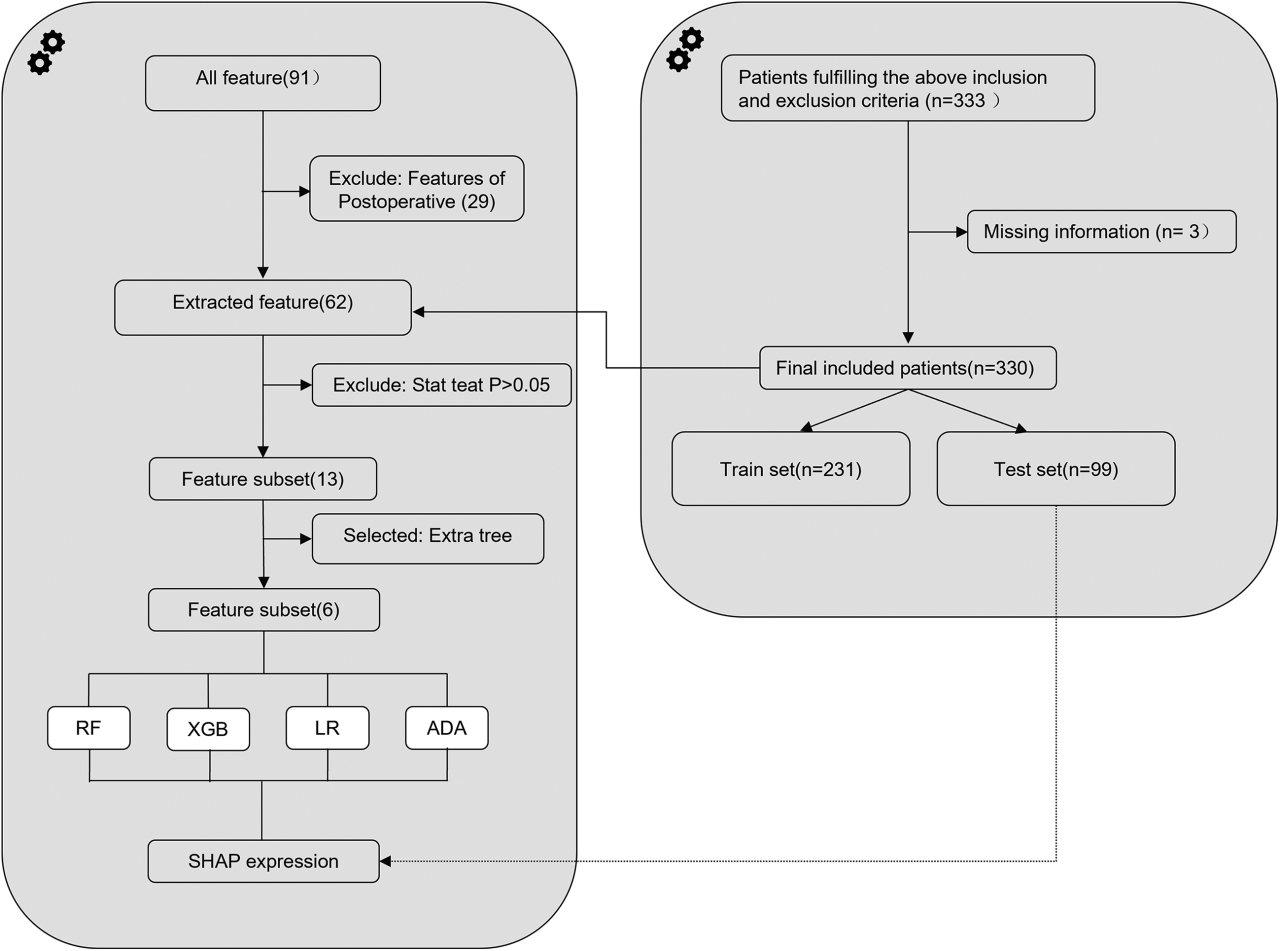
Flowchart depicting the design and analysis process of the machine-learning model for predicting decline in platelet count following interventional closure of patent ductus arteriosus in children. RF, random forest; XGB, extreme gradient boosting; LR, logistic regression; ADA, adaptive boosting; SHAP, SHapley Additive exPlanation.

**Figure 2:**
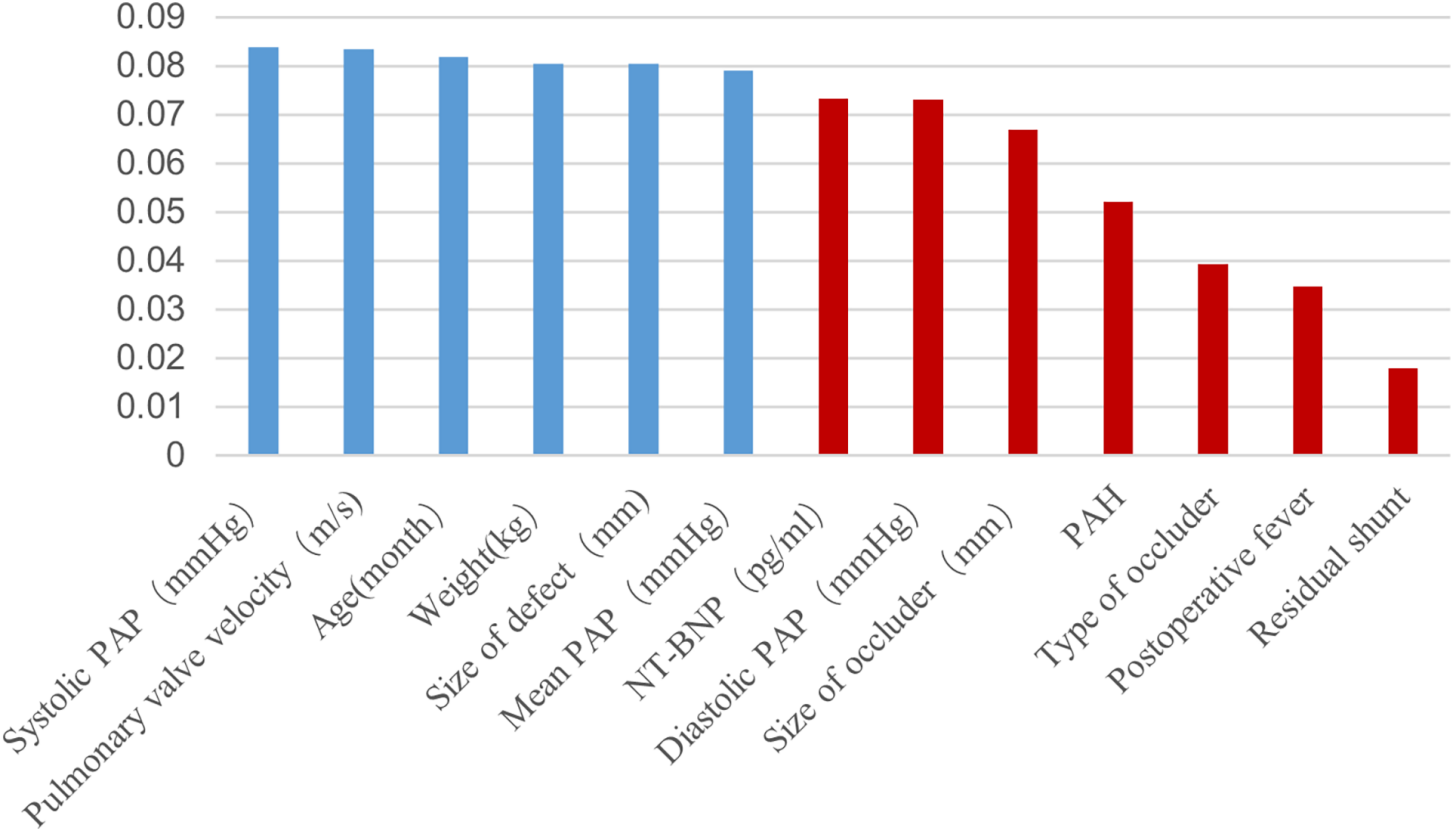
Extra tree selection predictive features. PAH, pulmonary arterial hypertension; PAP, pulmonary artery pressure.

The study database comprised 91 features, of which 62 preoperative and intraoperative features were selected as early predictive factors for post-intervention DPC in children with PDA undergoing transcatheter closure. Four ML models were established using these early predictive factors for early prediction, as illustrated in Figure 1. Data with a missing rate exceeding 20% were removed and relevant literature was systematically reviewed to identify potential factors to be considered. For variables with a missing proportion of less than 20%, the mode was used to estimate categorical variables and the mean was used to estimate continuous variables. Features with statistical significance (*P* < 0.05) in the univariate tests were initially selected to minimise potential overfitting caused by high-dimensional features and then extra tree algorithm was used to select low-dimensional features for model construction (14, 15). Ultimately, six features were chosen to construct the ML model (Figure 2).

### 2.4 Model construction and interpretation

The model construction involved the utilization of the following four supervised machine learning algorithms: logistic regression (LR), adaptive boosting (ADA), random forest (RF) and extreme gradient boosting (XGB). The models were evaluated using area under the curve (AUC) (16). Extra tree algorithm was applied to optimize the model parameters for each algorithm. SHAP was employed to interpret the ML models. Based on cooperative game theory, this method treats each feature variable in the dataset as a player and fairly allocates the cooperative gains by considering each player's contribution to the cooperative outcome, which, in this case, is the prediction result obtained by training the model. In this study, SHAP was applied to observe the effect of each feature on the prediction outcome during the prediction process (17).

### 2.5 Statistical analysis

In this study, we conducted data cleaning using Python (Anaconda distribution, version 3.8) with Pandas (version 1.5.3) and NumPy (version 1.23.5) libraries. Feature selection was performed using the extra tree algorithm, and base models including RF, XGB, LR, and ADA were built using the Scikit-learn package (version 1.2.1). Model interpretation was accomplished using SHAP. Statistical analysis was carried out with SPSS 26.00. Continuous variables with a normal distribution were presented as mean ± standard deviation and analyzed using independent sample *t*-tests. Non-normally distributed variables were described by quartiles and analyzed using the Kruskal-Wallis-test. Categorical variables were expressed as frequency proportions, and group differences were assessed using Chi-squared or Fisher's exact tests. Significance was defined as *P* < 0.05.

## 3 Results

### 3.1 Basic characteristics

A total of 330 children with PDA who underwent successful transcatheter closure were included in this study. Amongst them, 113 cases (34.2%) experienced DPC after the intervention, with 6 cases having an absolute platelet count <100 × 10^9^/L and 2 cases having an absolute platelet count <50 × 10^9^/L. Between the latter 2 cases, one exhibited skin bleeding and another showed skin and gingival bleeding. However, neither of them experienced visceral bleeding nor death. Table 1 presents the baseline characteristics of all children. In the NO-DPC group, the baseline platelet count was 305 × 10^9^/L, with a platelet count change of 39 × 10^9^/L. Meanwhile, in the DPC group, the baseline platelet count was 358 × 10^9^/L, with a platelet count change of 111 × 10^9^/L. The children who developed DPC after the intervention were younger, lighter in weight, had higher brain natriuretic peptide levels (NT-BNP) and had faster pulmonary valve velocities. Factors, such as size of defect, residual shunt, and pulmonary artery hypertension (PAH) were identified as risk factors for DPC. The differences between the two groups were statistically significant (*P* < 0.05).

**Table 1:** Baseline clinical characteristics of children.

	NO-DPC (<25% *n* = 217)	DPC (≥25% *n* = 113)	*P*-value
Characteristics of the children			
Age (m)	36 (24, 60)	25 (14, 36)	<0.001
Female, %	141 (65%)	79 (69.9%)	0.367
Weight (kg)	13.5 (11.0, 18.5)	11.0 (8.5, 14.5)	<0.001
Body mass index (kg/m^2^)	15.4 (14.0, 16.8)	15.3 (13.6, 17.1)	0.732
Length of hospital stay (day)	6.9 (6.0, 7.0)	6.9 (6.0, 7.4)	0.803
Pre-admission medications			
Aspirin, %	–	–	–
Heparin, %	–	–	–
Laboratory parameters (preoperative)			
Baseline platelet count (10^9^/L)	309 (266, 352)	348 (292, 421)	<0.001
Change in platelet count (10^9^/L)	39 (13.3, 65.8)	111 (93, 155)	<0.001
NT-BNP (pg/ml)	105.0 (63.5, 193.3)	174 (79, 484)	<0.001
Echocardiography (preoperative)			
Size of defect (mm)	2.4 (2.0, 3.0)	2.8 (2.1, 4.0)	<0.001
EF, %	69.8 ± 24.4	71 ± 24	0.327
Pulmonary valve velocity (m/s)	1.0 (0.9, 1.2)	1. 2 (1.0, 1.4)	<0.001
Left atrial diameter (mm)	25 (23, 28)	26 (24, 28)	0.813
Intraoperative			
Size of occluder (mm)	8 (6, 8)	8 (6, 10)	<0.001
Systolic PAP (mmHg)	28 (25, 33)	32 (28, 42)	<0.001
Diastolic PAP (mmHg)	16 (13, 19)	18 (14, 22)	0.001
Mean PAP (mmHg)	22 (19, 26)	25 (21, 33)	<0.001
PAH,%	59 (25.7%)	59 (52.2%)	<0.001
Residual shunt,%	10 (4.7%)	14 (12.4%)	0.011
Postoperative			
Postoperative fever, %	41 (18.9%)	33 (29.2%)	0.033
Postoperative bleeding, %	–	3 (2.7%)	0.039
Hemolysis, %	–	–	–

NO-DPC, no decline in platelet count; DPC, decline in platelet count; PAH, pulmonary arterial hypertension; PAP, pulmonary artery pressure; EF, left ventricular ejection fraction. *P*-value <0.05.

### 3.2 Model evaluation

**Figure 3:**
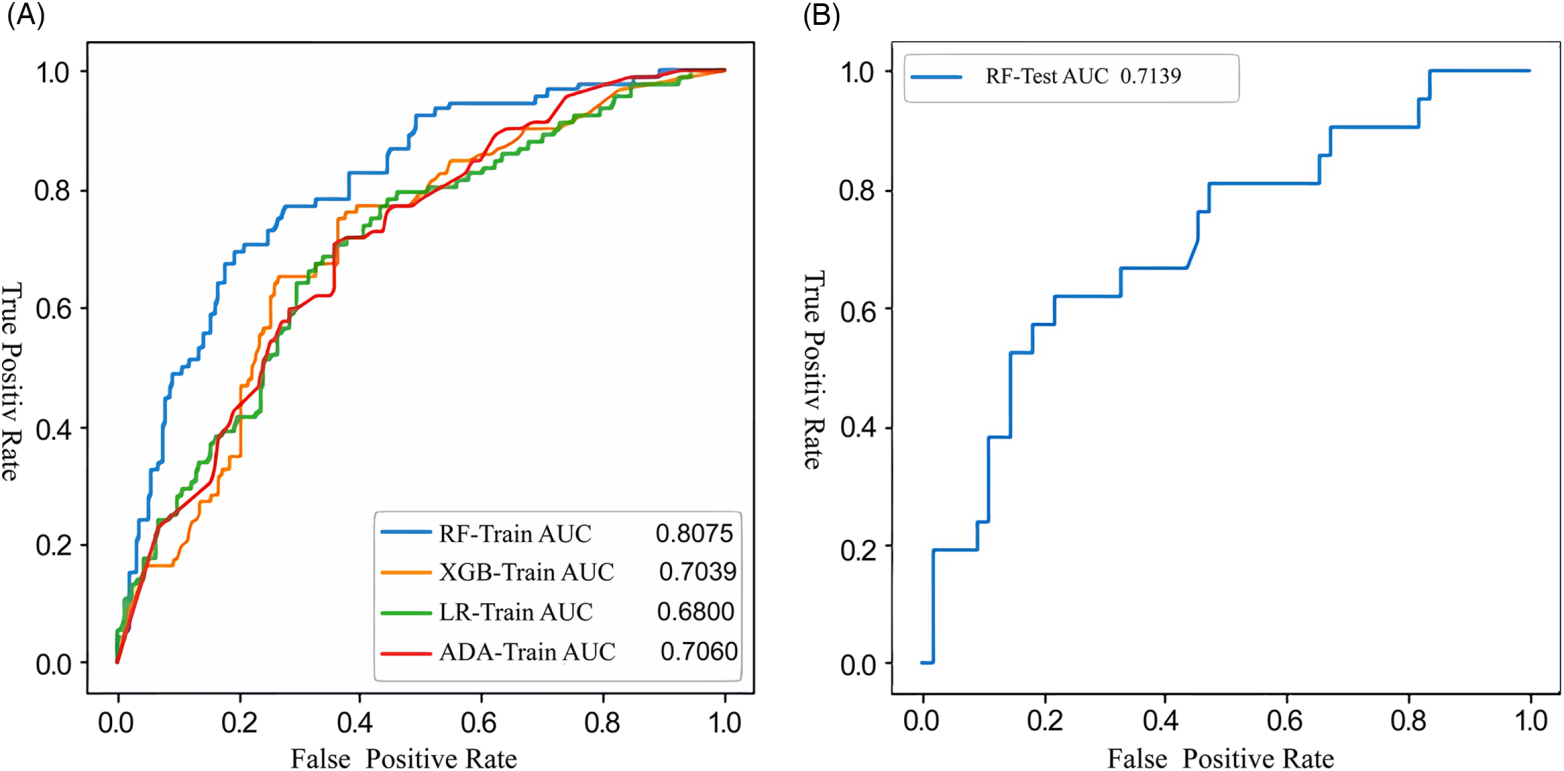
Receiver operating characteristic (ROC) curves illustrating the performance of the machine-learning model developed with the training **(A)** and testing **(B)** datasets for the prediction of DPC following interventional closure of PDA. RF, random forests; XGB, extreme gradient boosting; LR, logistic regression; ADA, adaptive boosting; DPC, decline in platelet count; PDA, patent ductus arteriosus.

The extra tree algorithm was used to select the top six features and four ML models were established to predict the occurrence of post-intervention DPC in children with PDA: RF, XGB, ADA and LR. The extra tree algorithm was utilized for the adjustment of model hyperparameters. The RF model demonstrated superior performance within the training dataset, as presented in Figure 3A. Furthermore, when applied to the test dataset, the RF model achieved an AUC value of 0.71, as illustrated in Figure 3B. Notably, this AUC value closely resembled that observed within the training dataset, signifying the absence of overfitting concerns. Consequently, the RF model was designated as the primary model for subsequent investigation within this study. The baseline clinical features of the training and testing sets are shown in Table 2.

**Table 2:** Baseline clinical characteristics of the training and testing sets.

	Total (*n* = 330)	Train (*n* = 231)	Test (*n* = 99)	*P*-value
Characteristics of the children				
Age (m)	35 (18, 48)	33 (16, 48)	31 (16, 48)	0.842
Female, %	110 (33.33%)	153 (66%)	66 (67%)	0.939
Weight (kg)	13 (10, 17)	13 (9, 18)	13 (9, 16)	0.884
Body mass index (kg/m^2^)	15.33 (13.94, 16.79)	15.38 (13.86, 17.3)	15.38 (14.1, 16.57)	0.453
Length of hospital stay (day)	6.9 (6.0, 7.0)	6.92 (6.00, 7.00)	6.96 (6.00, 7.00)	0.883
Pre-admission medications				
Aspirin, %	–	–	–	–
Heparin, %	–	–	–	–
Laboratory parameters (preoperative)				
Baseline platelet count (10^9^/L)	316 (273, 369)	317 (273, 373)	323 (274, 374)	0.884
Change in platelet count (10^9^/L)	65 (30, 98)	65 (24, 102)	53 (15, 94)	0.154
NT-BNP (pg/ml)	118 (67, 259)	107 (63, 266)	137 (73, 283)	0.228
Echocardiography (preoperative)				
Size of defect (mm)	2.5 (2.0, 3.2)	2.40 (1.90, 3.20)	2.60 (2.00, 3.25)	0.43
EF, %	70 (66, 74)	70 (66, 74)	71 (67, 75)	0.171
Pulmonary valve velocity (m/s)	1.0 (0.9, 1.2)	1.00 (0.90, 1.20)	1.00 (0.90, 1.20)	0.333
Left atrial diameter (mm)	25 (23, 28)	25 (23, 28)	25 (23, 27)	0.609
Intraoperative				
Size of occluder (mm)	8 (6,8)	8 (6, 8)	8 (6, 8)	0.957
Systolic PAP (mmHg)	29 (25, 35)	29 (25, 35)	30 (25, 36)	0.486
Diastolic PAP (mmHg)	17 (14,20)	16 (14, 20)	17 (14, 20)	0.484
Mean PAP (mmHg)	23 (19, 27)	22 (19, 27)	23 (19, 27)	0.641
PAH, %	118 (35.76%)	76 (33%)	42 (43%)	0.085
Residual shunt, %	24 (7.27%)	18 (7.9%)	6 (6.1%)	0.581
Postoperative				
Postoperative fever, %	74 (22.42%)	57 (25%)	17 (17%)	0.134
Postoperative bleeding, %	3 (0.91%)	3 (1.3%)	–	0.557
Hemolysis, %	–	–	–	–

NO-DPC, no decline in platelet count; DPC, decline in platelet count; PAH, pulmonary arterial hypertension; PAP, pulmonary artery pressure; EF, left ventricular ejection fraction. *P*-value < 0.05.

### 3.3 Feature importance of RF model

**Figure 4:**
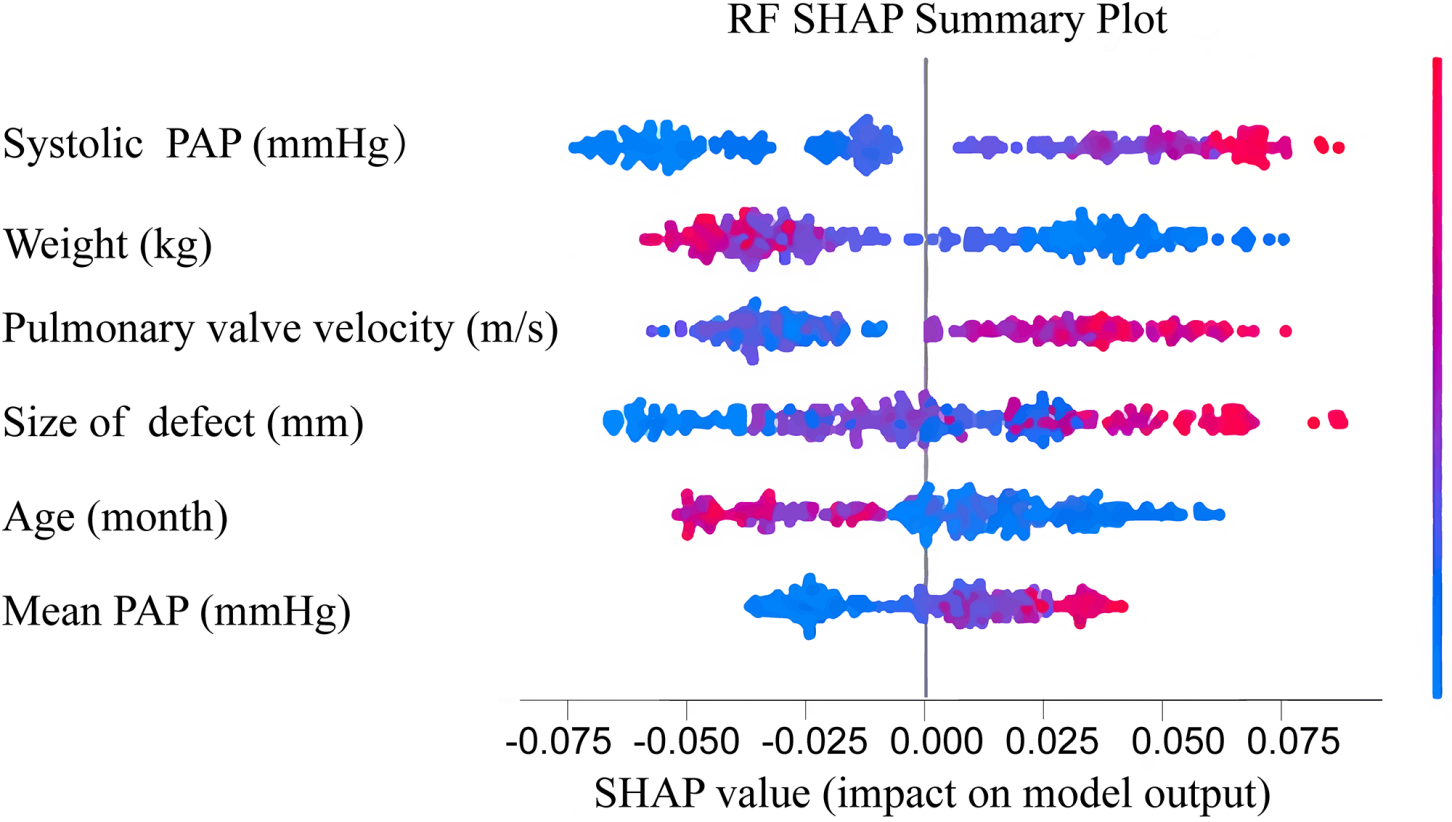
Feature importance ranked using sHapley additive exPlanation (SHAP) values in the RF model. Features are ranked based on the cumulative SHAP values across all individuals, representing the impact of each feature on RF model predictions. In the visualization, red denotes high feature values, while blue indicates low values. The *x*-axis represents the influence of SHAP values on model predictions. The higher the *x*-axis value, the greater the likelihood of DPC after interventional closure of PDA. PAP, pulmonary artery pressure; DPC, decline in platelet count; PDA, patent ductus arteriosus.

The importance scores of various features used to establish the RF model for early prediction of post-intervention DPC in children with PDA were calculated (Figure 4). The *Y*-axis represents the feature importance. These features included systolic pulmonary artery pressure (PAP), size of defect, weight, mean PAP, pulmonary valve velocity and age. Amongst them, systolic PAP, weight and pulmonary valve velocity ranked as the top three in terms of importance. SHAP was applied in this study to gain further insights into the significance of these features. SHAP values can help understand the individual effect of each feature on the model. In Figure 4, the magnitude of the SHAP values directly corresponds to the extent of their impact on the model. The *X*-axis within the figure portrays the SHAP values, color-coded on a spectrum from blue to red, symbolizing low to high SHAP values, respectively. For instance, patients with higher systolic PAP (depicted as red dots on the graph) and lower weight (shown as blue dots on the graph) are more prone to post-intervention DPC. Similarly, children with larger size of defect, younger age, and higher pulmonary valve velocity are more likely to develop post-intervention DPC.

### 3.4 SHAP values of individual prediction for interpretation

**Figure 5:**
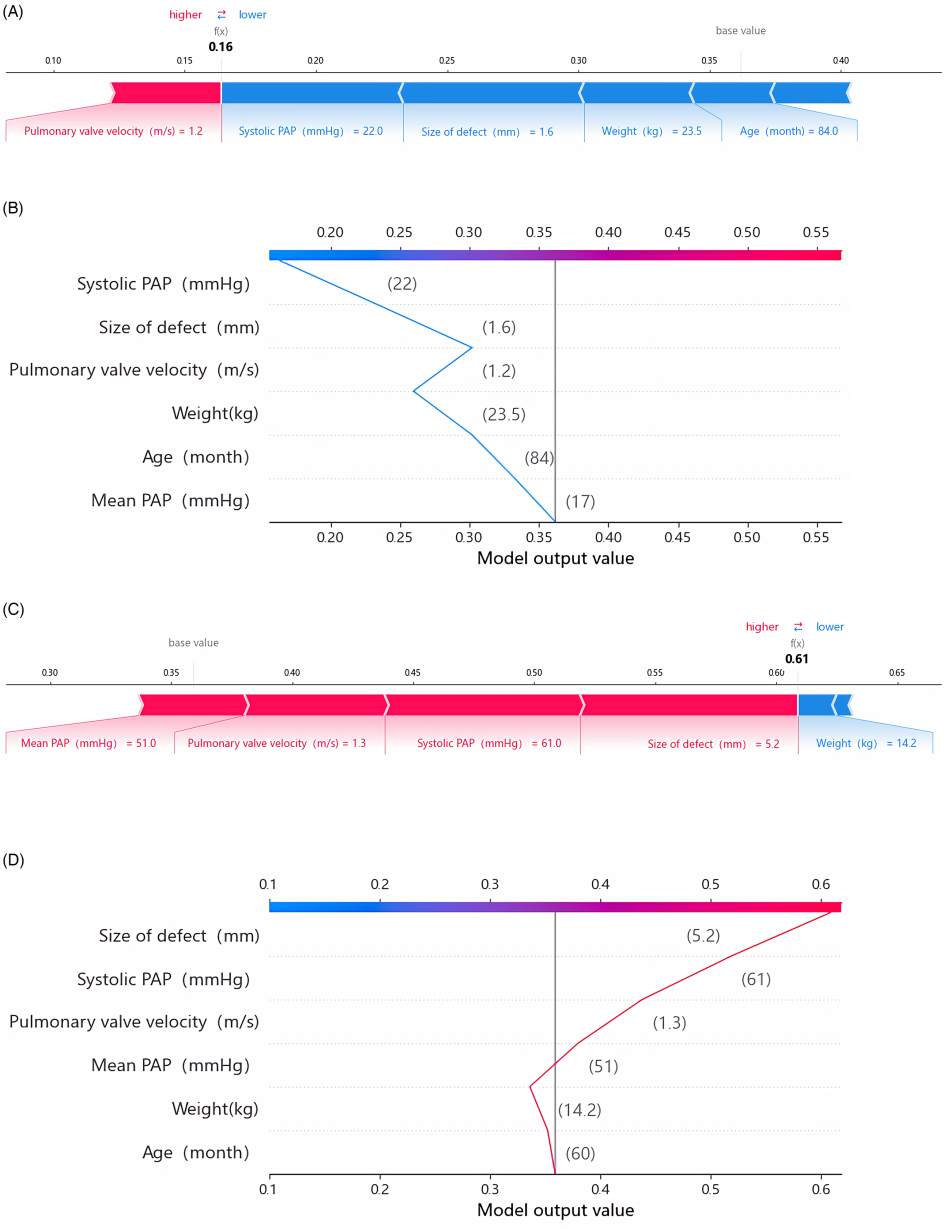
SHAP explanation force plots **(A)** and decision plot **(B)** of patient No. 1 (NO-DPC). SHAP explanation force plots **(C)** and decision plot **(D)** of Patient No. 2 (DPC). The force plots illustrate the individual feature contributions to class classification (prediction paths). The decision plot demonstrates how each feature contributes to the transition of the decision score from the base value to the classifier's predicted value. PAP, pulmonary artery pressure.

In this investigation, SHAP was employed to elucidate predictions for both the entire cohort and individual children, thus enhancing our understanding of the forecasted outcomes. For the two children correctly predicted in this study, SHAP was applied to interpret the prediction model and results. For child 1, who did not experience DPC, the model predicted a low likelihood of post-intervention DPC (Figures 5A,B). Meanwhile, for child 2, who experienced DPC, the model predicted a high likelihood of post-intervention DPC (Figures 5C,D).

Child 1 was a male admitted at 36 months of age, with a weight of 14.7 kg and a pre-intervention platelet count of 471 × 10^9^/L. Echocardiography revealed a 2 mm size of defect and a pulmonary valve velocity of 0.8 m/s. During the procedure, the catheter-measured systolic PAP was 23 mmHg and the mean PAP was 18 mmHg. The patient underwent successful PDA closure with a 4–6 mm ADO occluder, resulting in no residual shunt. However, a decline of 18% in platelet count was observed compared with the pre-procedure levels. The RF model predicted a risk of 0.16 for post-intervention DPC for this patient, with systolic PAP, size of defect, weight and pulmonary valve velocity showing significant contributions to the model.

Child 2 was a male admitted at 60 months of age, with a weight of 14.2 kg and a pre-intervention platelet count of 220 × 10^9^/L. Echocardiography revealed a 5.2 mm size of defect, a pulmonary valve velocity of 1.3 m/s and a moderate-to-severe PAH. During the procedure, the catheter-measured systolic PAP was 64 mmHg and the mean PAP was 51 mmHg, both indicating moderate-to-severe PAH. The child underwent PDA closure with a 14 mm ADO occluder, resulting in residual shunt with a flow velocity of 2.4 m/s. The post-intervention platelet count reached a minimum value of 31 × 10^9^/L, accompanied by bleeding in the gums and skin. However, no visceral bleeding was observed. After treatment with vitamin K injections, haemostatic agents and vitamin C injections, the platelet count recovered to 64 × 10^9^/L after 10 days. The RF model predicted a risk of 0.61 for post-intervention DPC for this child, with size of defect, systolic PAP, pulmonary valve velocity and mean PAP showing significant contributions to the model. In actuality, this patient experienced severe platelet decline, decreasing by 85.9% compared with the pre-procedure baseline value.

## 4 Discussion

This study aimed to develop an ML model to early predict the occurrence of DPC after intervention closure in children with PDA. Comparison of four ML models showed that the RF model performed the best and could more accurately predict post-intervention DPC. SHAP was applied for interpretation to further understand this ML model. The results revealed that the top six ranked features in the RF model were systolic PAP, size of defect, weight, mean PAP, pulmonary valve velocity and age. Larger systolic PAP, mean PAP, size of defect and pulmonary valve velocity were associated with a higher risk of post-intervention DPC, whereas older age and heavier weight were associated with a lower likelihood of DPC.

Post-intervention DPC in children with PDA has drawn clinical attention but the underlying mechanisms remain unclear. A survey involving 299 patients with congenital heart disease found that 135 of them experienced platelet decline, including 10 cases of severe decline (<50 × 10^9^/L), with two cases exhibiting major bleeding. However, all patients survived. Further analysis suggested that size of occluder, residual shunt, mean PAP and age were independent risk factors for post-intervention DPC (18). Another study involving 1,581 patients with PDA confirmed size of defect and residual shunt as independent risk factors for post-intervention DPC (3). Zhou et al. found that amongst 336 patients with congenital heart disease, 21 experienced severe platelet decline and the size of occluder and post-intervention residual shunt were independent influencing factors. Additionally, bone marrow puncture was performed on four patients with platelet count <50 × 10^9^/L after congenital heart disease intervention closure, showing that the mechanism of DPC was due to excessive platelet consumption rather than decreased platelet production (19). Although the risk of post-intervention DPC in children with congenital heart disease is low, the severity of such decline may lead to significant bleeding, making early prediction crucial. Currently, there are no reports on whether early intervention for thrombocytopenia can significantly improve clinical prognosis. Future research should focus on multi-center, large-scale prospective studies to further validate the impact of early prediction and intervention for thrombocytopenia on clinical outcomes.

In recent years, ML models have played an essential role in disease prediction (20). However, for clinicians, understanding how to establish ML models and how these features affect the model's decision-making process remain unclear. Hence, explaining ML models is crucial for clinical practitioners (8, 21). In the present study, SHAP was used to interpret the RF model. Based on game theory, this method calculated the SHAP values for each feature and explained their effect on the model (9, 17). The results showed that systolic PAP, mean PAP and size of defect had a positive correlation with the RF model, consistent with previous research findings. Additionally, age and weight had a negative correlation with the RF model. Whilst previous studies suggested that older age was associated with a higher risk of post-intervention DPC (18), the present study focused on a paediatric population and children with younger age and lighter weight may undergo more significant surgical trauma, thus increasing the likelihood of DPC. Moreover, a faster pulmonary valve velocity increased the risk of post-intervention DPC, which has not been reported in previous studies. A higher pulmonary valve velocity may indicate faster pulmonary artery blood flow, leading to increased mechanical consumption of platelets upon contact with the occluder. Furthermore, previous studies showed that residual shunt is an independent risk factor for post-intervention DPC (3, 19). In the present study, residual shunt was not included in the model construction due to the limited sample size and the low number of children with residual shunt. As a result, it was not considered in the analysis. Further studies with a larger sample size are needed to investigate its role in post-intervention DPC. The present study provided explanations for two correctly predicted children, as well as an explanation for constructing the RF model, offering an enhanced understanding of the decision-making process and the effect of features for individual predictions.

However, this study has some limitations. Firstly, it is a single-centre study with data from only 330 children, necessitating the inclusion of more cases from multiple centres to construct and validate the model. Secondly, although the predictive capability of the optimal model was satisfactory, external validation using an independent cohort is still needed before clinical application. Lastly, post-intervention DPC was examined only in children with PDA and interventions for other types of congenital heart disease were not investigated.

## 5 Conclusion

In conclusion, an ML model was established to predict the risk of post-intervention DPC in children with PDA. SHAP was used to interpret the model, revealing the significant effect of systolic PAP, size of defect, weight, mean PAP, pulmonary valve velocity and age on the model's performance. The research findings are valuable for clinical practitioners to early predict whether children with PDA would experience DPC after intervention, enabling timely intervention measures.

## Data Availability

The raw data supporting the conclusions of this article will be made available by the authors, without undue reservation.
